# Use of in vivo induced antigen technology to identify genes from *Aeromonas salmonicida* subsp. *salmonicida* that are specifically expressed during infection of the rainbow trout *Oncorhynchus mykiss*

**DOI:** 10.1186/s12917-014-0298-0

**Published:** 2014-12-14

**Authors:** Simon Menanteau-Ledouble, Hatem Soliman, Gokhlesh Kumar, Mansour El-Matbouli

**Affiliations:** Clinical Division of Fish Medicine, Department for Farm Animals and Veterinary Public Health, University of Veterinary Medicine, Veterinarplatz 1, Vienna, 1210 Austria; Fish Medicine and Managements, Department of Animal Medicine, Faculty of Veterinary Medicine, Assiut University, 71515 Assiut, Egypt

**Keywords:** IVIAT, Antigenic profile, UDP deacetylase, RpoD, TonB, Virulence, Transcription levels

## Abstract

**Background:**

*Aeromonas salmonicida* is a major fish pathogen associated with mass mortalities in salmonid fish. In the present study, we applied In Vivo Induced Antigen Technology (IVIAT), a technique that relies on antibodies adsorbed against in vitro cultures of the pathogen, to a clinical isolate of *A. salmonicida* subsp. *salmonicida*.

**Results:**

The results from IVIAT allowed identification of four proteins that were upregulated in the fish samples: A UDP-3-O-acyl-N-acetylglucosamine deacetylase, an RNA polymerase sigma factor D as well as TonB and a hypothetical protein. Subsequent investigations were performed using real-time PCR and cDNA synthesised from infected spleen, liver and anterior kidneys. These confirmed that the transcription level of each of these genes was significantly upregulated during the infection process compared to bacteria in vitro.

**Conclusions:**

The present studied identified four genes that were upregulated during the infectious process and are likely to play a role in the virulence of *A. salmonicida*. Because these are antigenic they might constitute potential targets for the development of new vaccine as well as therapeutic agents.

## Background

*Aeromonas salmonicida* is a Gram-negative psychrophilic aquacultural pathogen that has the ability to infect a variety of fish species but is mostly an issue for the salmonid industry. In these species, it is the causative agent of a condition often referred to as “furunculosis”, based on the characteristic furuncles that can appear on the dermis of the fish and can extend deep into the musculature [1]. The disease can take several forms, ranging from peracute to more chronic [2] and a number of virulence factors have previously been described in *A. salmonicida*. These include adhesion factors such as the A-layer [3] and pili of both the type I [4] and IV [5]. In addition, multiple extracellular proteins have been described including the P1 and P2 proteases, caseinase and metallo-caseinase [6] and the lipase GCAT [7]. However, studies by Vipond et al. [8] suggest that several of these extra-cellular products are not required for the virulence of the bacterium. In addition, a type III secretory system [9] has been described which now seems to play a central role in the bacterium’s virulence [10-12]. Notably, this type III secretion system is inhibited at temperatures superior to 25°C [13,14], which may have delayed its discovery. Nonetheless, despite these advances, our understanding of the virulence mechanisms in *A. salmonicida* is not yet complete.

Several vaccines have been developed against *A. salmonicida* and are now available to the industry. In particular, an oil-adjuvanted injectable solution was introduced in the early 1990ies and has been widely adopted by the industry [15]. This vaccine is considered very efficacious and is sometime credited for reducing the incidence of furunculosis in Scotland and Norway. However, a subsequent study by Smith and Hiney [16] has suggested that the reported decrease in the incidence of infections by *A. salmonicida* might actually predate the introduction of the vaccine in these countries. Moreover, intra-peritoneal injection is (according to Horne, 1997 as cited by Plant and Lapatra [17]) an impractical delivery method on smaller fish which leaves the earliest stage of production unprotected. Furthermore, the development of intraperitoneal lesions following injection of oil-adjuvanted vaccines have been reported [18-20]. Alternative methods of delivery by immersion and oral vaccination have encountered limited success: the protection they confer is not considered to be as strong or long lasting as the one delivered by IP injection [21].

First suggested in 2000 [22], In vivo induced antigen technology (IVIAT) is still a recent technique. It relies on harvesting serum or plasma from individuals exposed to the pathogen of interest, adsorbing the antibodies it contains against an in vitro culture of the organism before using these adsorbed antibodies to screen an expression library of the pathogen [22,23]. Because of this preliminary adsorption step, IVIAT allows identifying antigens that are specifically expressed during the infection process. These antigens correspond to molecules that are not expressed under normal culture conditions and therefore difficult to study. For this reason, IVIAT offers unique insights in the pathogen’s virulence as well as suggests promising targets for the development of vaccines and new therapeutic agents.

In *A. salmonicida*, it has been shown that a number of virulence factors were differentially expressed based on environmental conditions. For example, the Flp/Fap pilin proteins [24], the enzyme enolase [25] as well as a number of molecules of the iron acquisition apparatus [26] have all been shown to be upregulated under iron-deprived culture conditions.

The aim of the present study was therefore to apply IVIAT to identify genes that are specifically expressed by *A. salmonicida* subsp. *salmonicida* during infection of the rainbow trout host. Four genes were identified in such way. Furthermore, alongside the screening procedure proposed by Handfield et al. [22], a series of RT-qPCR was performed that aimed to compare the transcription levels of these genes between the bacteria cultivated in vitro or in infected fish.

## Results

### Preparation of the antibodies

Fish were infected with *A. salmonicida* subsp. *salmonicida* through a variety of routes and blood was sampled at various time points. Then, the plasmas that contained antibodies reactive against *A. salmonicida* were pooled and adsorbed against in vitro cultures of *A. salmonicida* following the procedure described in Absoption of the antibodies section. To test the efficiency of this adsorption process, both pre-adsorbed and adsorbed sera were used in western-blots against: lysates from both *A. salmonicida* and *E. coli* were incubated against both the pre-adsorbed and adsorbed sera and reactive antibodies were detected by colorimetry.

**Figure 1:**
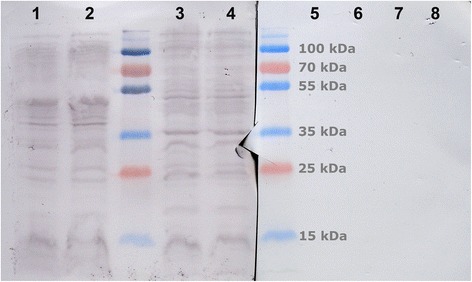
**Western-blot on lysates from** ***Aeromonas salmonicida*** **subsp. s** ***almonicida*** **isolate A14390 and** ***Escherichia coli*** **XL1-Blue MRF’.** In order to assess the efficiency of the adsorption process, lysates from in vitro cultivated bacteria were analyse by Western-Blot. The results confirmed that the antibody solution contained reactive antibodies that were removed during the adsorption process. Line 1: *Aeromonas salmonicida* subsp. s*almonicida* isolate A14390, whole cells; non-adsorbed antibodies. Line 2: *Aeromonas salmonicida* subsp. s*almonicida* isolate A14390, sonicated cells; non-adsorbed antibodies. Line 3: *Escherichia coli* XL1-MRF’, whole cells; non-adsorbed antibodies. Line 4: *Escherichia coli* XL1-MRF’, sonicated cells; non-adsorbed antibodies. Line 5: *Aeromonas salmonicida* subsp. s*almonicida* isolate A14390, whole cells; adsorbed antibodies. Line 6: *Aeromonas salmonicida* subsp. s*almonicida* isolate A14390, sonicated cells; adsorbed antibodies. Line 7: *Escherichia coli* XL1-MRF’, whole cells; adsorbed antibodies. Line 8: *Escherichia coli* XL1-Blue MRF’, sonicated cells; adsorbed antibodies.

As expected, the non-adsorbed fish antibodies recognized multiple antigens within both bacterial lysates (Figure 1). On the contrary, the adsorbed fish antibodies failed to react with either bacterial lysates.

### Screening of the library

After confirmation of the efficiency of the adsorption process, the adsorbed antibodies were used to screen an expression library of *A. salmonicida* subsp. *salmonicida* as described in paragraph 5.7. A total of 12074 plaque forming units expressing sequences belonging to genes of the *A. salmonicida*’s genome were screened during the project and this screening generated a total of four confirmed positive signals. Based on sequence homology (Table 1), these genes were identified as an Uridine diphosphate (UDP) 3-O-acyl-N-acetylglucosamine deacetylase membrane peptidase [GenBank: ABO88583], the RNA polymerase sigma factor D (RpoD) [GenBank: ABO91429], a hypothetical protein [GenBank: ABO92001] as well as TonB [GenBank: ABO89943].

**Table 1:** **List of the genes identified in this study**

**Sequence identified**	**GenBank accession number**	**Percentage of identity**
UDP 3-O-acyl-N-acetylglucosamine deacetylase membrane peptidase	ABO88583	100%
RNA polymerase sigma factor D	ABO91429	76%
Hypothetical protein	ABO92001	100%
TonB	ABO89943	95%

### Real time quantitative PCR

**Figure 2:**
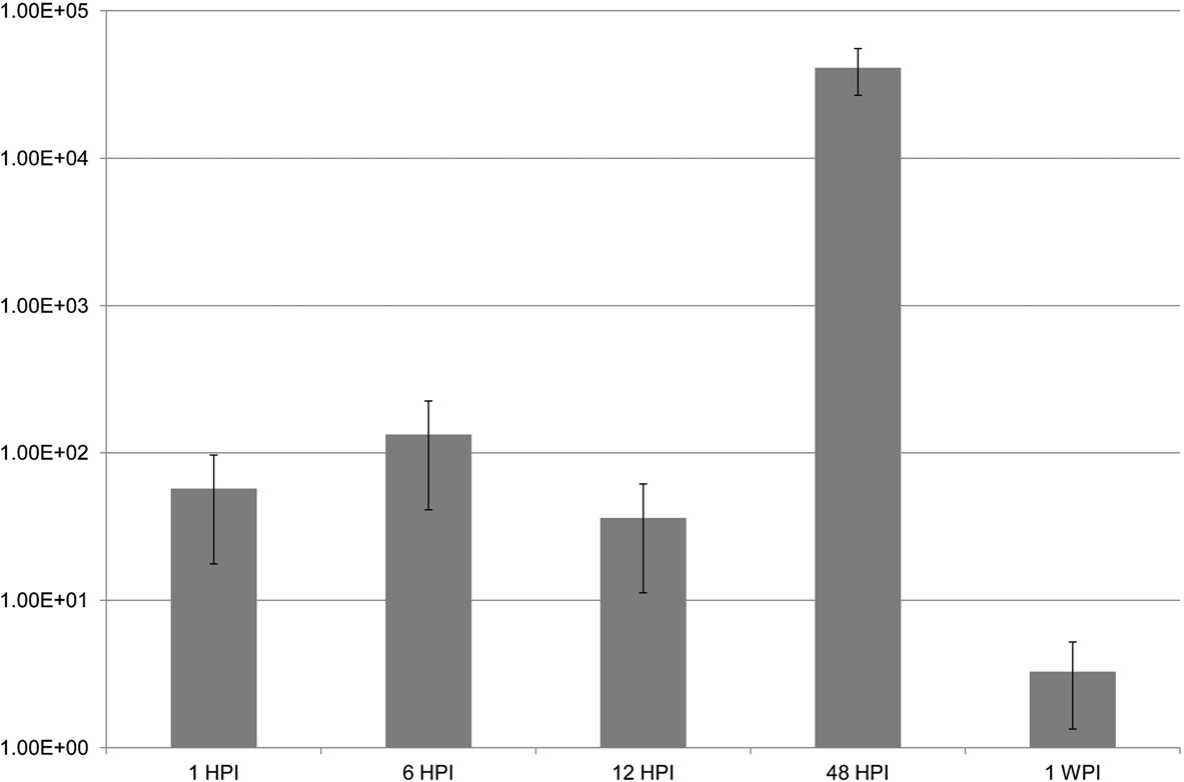
**Relative abundance of** ***Aeromonas salmonicida*** **subsp. s** ***almonicida*** **in spleen of rainbow trout at various time-points.** The relative bacterial number was calculated by comparing the number of copies of bacterial 16S rRNA to that of the host’ β-actin. The fish had been infected intra-peritoneally with a solution of 10^3^ CFU of *A. salmonicida* in 0.9 M phosphate buffered saline. Fish were sampled at 1, 6, 12, 48 hours and 1 week post-infection. Each value represents the mean of triplicate independent biological samples and error bars indicate standard deviation.

A series of RT-qPCR was performed in order to determine the time points at which the bacterial loads were highest in the infected organs (Figure 2). These corresponded to 6 and 48 hours post-infection (HPI).

Additional RT-qPCRs were then conducted to calculate the mean fold change, between the infected organs and in vitro cultures, in the expression of the genes coding for the four proteins identified through the screening of the library.

**Figure 3:**
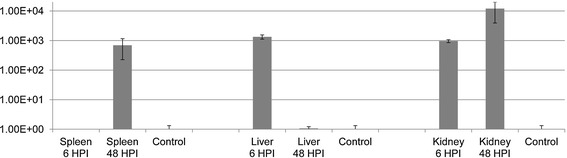
**Change in the transcription of the gene coding for the UDP-3-O-acyl-N-acetylglucosamine-deacetylase investigated during this study.** The relative gene expression profile of the gene coding for UDP-3-O-acyl-N-acetylglucosamine deacetylase was calculated in the spleen, liver and anterior kidney samples of rainbow trout, *Oncorhynchus mykiss* infected with *A. salmonicida* sampled 6 and 48 hours post infections. qPCR data were normalized against 16S rRNA expression. Relative gene expression changes were determined by calculating the mean expression values from the control and infected spleen, liver and anterior kidney samples of fish according to the 2^-ΔΔC^T method. Each value represents the mean of triplicate independent biological samples and error bars indicate the confidence intervals.

The expression of the gene coding for the UDP-3-O-acyl-N-acetylglucosamine deacetylase membrane peptidase was not detectable in the spleen at 6 HPI. However, in this organ, it was significantly upregulated at 48 HPI compared to the in vitro cultures (Figure 3). In contrast, it was detectable in the liver at both 6 and 48 HPI. However, at 48 HPI the mean fold difference of the gene expression in the liver compared to the bacteria in vitro was only of 1.07, a difference that was not statistically significant (p value of 0.83 as calculated by T-Test). In the anterior kidney, this gene was significantly upregulated at both time points and in average the mean fold change in gene expression between infected fish samples and in vitro cultures was 3.28E^+03^ ± 1.77E^+03^, as calculated using the 2^-ΔΔ*C*^_T_ method (Figure 3).

**Figure 4:**
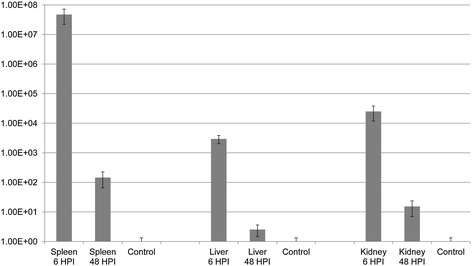
**Mean fold change in the expression of the** ***rpoD*** **gene investigated during this study.** The relative gene expression profile of RpoD was calculated in the spleen, liver and anterior kidney samples of rainbow trout. Normalization was performed against 16S rRNA expression as described in Figure 3.

The expression of the RNA polymerase sigma factor *rpoD* was detectable both at 6 and 48 HPI in all three organs. In each case, it was significantly upregulated compared to the in vitro cultures (Figure 4). The mean fold change in gene expression between infected organ samples and in vitro cultures was 7.86E^+06^ ± 5.03E^+06^.

**Figure 5:**
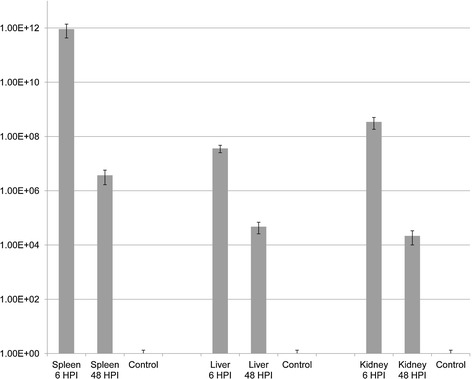
**Mean fold change in the expression of the gene coding for the hypothetical protein investigated during this study.** The relative gene expression profile of the gene coding for the hypothetical protein was calculated in the spleen, liver and anterior kidney samples of rainbow trout. Normalization was performed against 16S rRNA expression as described in Figure 3.

Similarly, the gene coding for the hypothetical protein was significantly upregulated in all organs samples at both time points with a mean fold change of 1.51E^+11^ ± 9.63E^+10^ (Figure 5).

**Figure 6:**
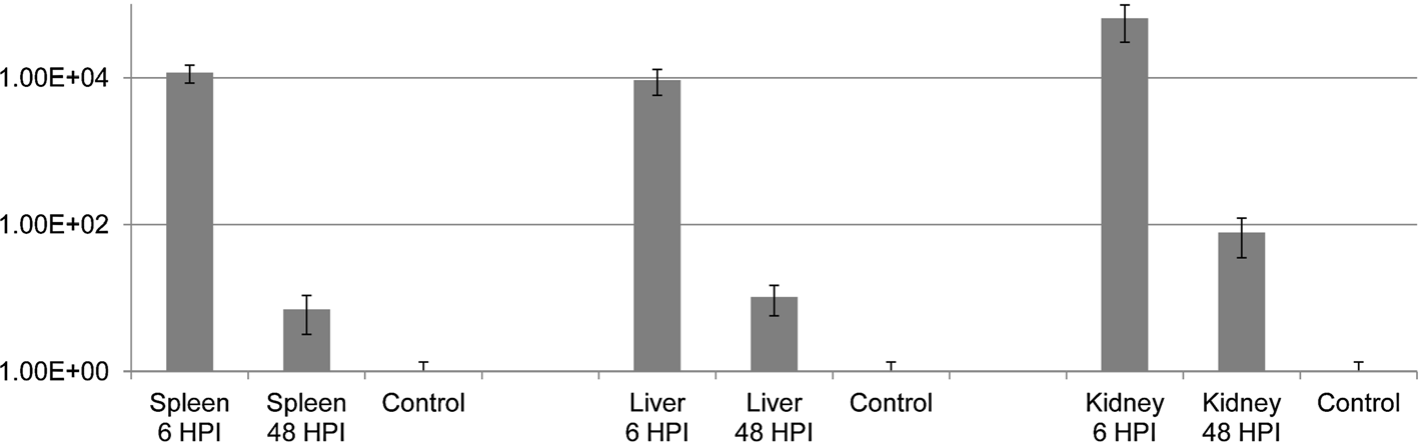
**Mean fold change in the expression of the** ***tonB*** **gene investigated during this study.** The relative gene expression profile of the TonB gene was calculated in the spleen, liver and anterior kidney samples of rainbow trout. Normalization was performed against 16S rRNA expression as described in Figure 3.

Finally, this was also the case of *tonB* for which a mean fold change of 1.41E^+04^ ± 5.72E^+03^ was calculated (Figure 6).

In the qPCR, the C_T_ values obtained from the negative controls, both uninfected fish samples and no-template controls were either non-detectable or above 34. This lack of amplification in the negative controls confirmed the specificity of our primers for the genes of interest.

## Discussion

The library was constructed by enzymatic digestion of the genome of *A. salmonicida* subsp. *salmonicida*. This allowed for a direct and efficient ligation of the fragments inside the pre-digested vector. However, because all restriction sites are not equally distributed through the genome, it is likely that such enzymatic digestion introduces a bias in the distribution of the genes through the library [23,27].

### Evaluation of the bacterial abundance

Bacterial numbers in the spleen were highest between 6 and 48 HPI (Figure 2). Presumably, this was the time at which the highest number of bacteria had made their way to the spleen and before the host’s immune system had ramped-up to clear away the infection. It is worth mentioning again that the challenge doses were purposely low, to minimize the risk of the bacteria killing the fish. In the case of a lethal infection, the kinetic of the bacterial distribution is likely to be different.

### Genes identified during this study

UDP-3-O-acyl-N-acetylglucosamine deacetylase is a membrane peptidase involved in the second step of the synthesis of lipid A [28]. This step is the first thermodynamically favourable one and commits the substrate to the synthesis [29]. Lipid A serves as the anchoring region for the lipopolysaccharide (LPS) chain [30], a molecule that helps the bacteria evade the immune system, and in particular the complement cascade [31]. UDP-3-O-acyl-N-acetylglucosamine deacetylase was identified through our plate screening, suggesting that this molecule was more expressed during the infection process. However, when qPCR was performed, expression of the corresponding gene could not be detected in the spleen at 6 HPI, possibly due to the lower number of bacteria in the samples at that time-point. At 48 hours, only limited difference was detected between the transcription level of the gene in the infectious samples and the in vitro cultures. Furthermore, the mean fold change in gene expression in the liver, as calculated by the 2^-ΔΔCt^ method, was found not to be statistically significant (1.07 ± 1.56E^−01^). This was unusual as the other genes investigated were all found to be upregulated in all three organs and at both time points investigated, compared to bacteria cultivated in vitro. In average, across all organs and time-points, the protein was significantly upregulated with a mean fold change in the expression of the gene coding for the UDP-3-O-acyl-N-acetylglucosamine deacetylase was 3.28E^+03^ (±1.77E^+03^). Interestingly, previous work carried out by Sorensen [32] showed that in *E. coli* an increase in the availability of UDP-3-O-acyl-N-acetylglucosamine deacetylase was not correlated to a significant increase in the levels of the corresponding messenger RNA. The authors hypothesized that the augmented production of this protein was achieved either by an increase in the translation levels of the existing mRNA or by a slowing down of the turnover of the existing proteins [32]. If a similar mechanism exists in *A. salmonicida*, it would explain how the protein could be more present during the infection process, as suggested by the result of the library screening, while being only moderately upregulated. However, more research will be required to answer that question definitively. UDP-3-O-acyl-N-acetylglucosamine deacetylase was also unusual in that it is the only gene that was found to be more upregulated at 48 HPI than at 6 HPI (in the spleen and kidney). The reason for this discrepancy is not known, it is possible that it reflects a progression in the infection process between these two time-points. In this case, the other genes might be involved in the immediate adaptation and survival to the conditions inside the host while UDP-3-O-acyl-N-acetylglucosamine deacetylase would be part of a later response. However, this hypothesis will require a more detailed analysis of the differential expression of the bacterial genes to be investigated.

The second gene identified through IVIAT was a RNA polymerase sigma factor RpoD. *rpoD* encodes for the major sigma factor σ^70^ [33,34] and is involved in the transcription/translation process necessary for normal cell survival [33]. In aeromonads, *rpoD* has received extensive scrutiny regarding its potential as a tool for phylogenical studies [35,36] but seems to have garnered little interest otherwise. While *rpoD* is regarded as a housekeeping gene, its expression has been shown to be variable in several organisms including *Vibrio salmonicida* [37]. In *Campylobacter jejuni* [33], it has been shown that *rpoD* displays a stress-dependant induction of transcription. Furthermore, in the pathogen *Xylella fastidiosa*, transcriptional profiling has shown that *rpoD* was part of the *rpfF* regulon alongside a number of virulence factors such as that involved in the secretion of the pillus apparatus [38]. Taken together, these findings appear to suggest that *rpoD* might be part of the adaptive response of some bacterial pathogens to stressors. Moreover, the list of these stressors appears to include some that the bacterium encounters upon infecting its host. This hypothesis would be consistent with the present findings that shows that *rpoD* was upregulated during infection in rainbow trout compared to the in vitro cultures (mean fold change in gene expression: 7.86E^+06^). Finally, *rpoD* appears more transcribed earlier in the infection process (mean fold change: 4.32E^+07^ ± 2.68E^+07^ between the 6 HPI and 48 HPI samples), which would be consistent with it being an adaptive response to the entry into the host. It would therefore be of interest to investigate the expression levels of this protein under a variety of culture conditions to confirm that it indeed responds to environmental stressors.

The present study also identified a hypothetical protein. Both the antibody screening and the qPCR analysis (mean fold change in gene expression: 1.51E^+11^) suggested that the protein was more expressed during the infection process in fish. However, its role is unknown and will require more research to determine. Targeted deletion mutants, for example, followed by a challenge might allow determining how important a role this protein plays in virulence.

Finally, the last protein identified was TonB. The corresponding gene was confirmed as being upregulated during the infection process with a mean fold change in gene transcription levels of 1.41E^+04^ (±5.72E^+03^) between infected fish organs and in vitro cultures. TonB is a well conserved protein and acts by bridging the periplasmic space and transferring the energy produced by the proton motive force from the cytoplasmic membrane to proteins on the outer-membrane [39]. This allows to provides energy for the uptakes of nutrients and complexes that are either too large to pass through the porin molecules (molecular weight above 600 Da) or that need to be transferred against the concentration gradient [40]. In particular, TonB is well known for its role in the uptake of Vitamin B_12_ and iron [40,41] either through the uptake of siderophores or that of heme. Because iron plays an important factor in bacterial growth during the infection process, iron acquisition is an important part of bacterial virulence, including in aeromonads and vibrionaceae [30,42]. In *A. salmonicida*, iron deprivation has been shown to stimulate both the production of a catechol-type sideropohore [24] as well as that of a heme-binding protein on the outer-membrane [26] and it is likely that iron acquisition mechanisms are required for the growth of bacteria during the infectious process [30]. It is worth noting that the range of substrate targeted by TonB-dependent mechanisms is not limited to iron and vitamin B_12_ and include such nutrients as nickel as well as maltodextrins or sucrose [41]. In addition, a molecule presenting a strong homology to TonB has also recently been reported to be required for the secretion of the extotoxin aerolysin in *Aeromonas hydrophila* [43]. It is therefore not surprising that TonB would be more expressed during the infection process, as described in the present study.

By its design, IVIAT focuses on antigens that are specifically expressed in vivo. However, for most bacteria, it is expected that additional antigens exist that are expressed under both in vivo and in vitro conditions. In the case of *A. salmonicida*, the existence of such in vitro expressed antigens is demonstrated by the protective effect of the bacterin vaccine [18,21,44]. For example, polysaccharide extracted from the supernatant of broth cultures of *Aeromonas salmonicida* has been shown to be immunogenic [45]. Nonetheless, application of IVIAT can still help us to identify other immunogenic components that are not part of the vaccines currently used by the industry. These antigens might constitute new targets for the development of complementary vaccinal solutions. In particular, a vaccine able to generate a stronger response after being administered orally or by immersion would allow protecting fish before they reach an age at which an intra-peritoneal vaccination is practical.

## Conclusions

The present findings broaden our understanding of the complex virulence mechanisms of *A. salmonicida*. Additional studies, using lethal doses of bacteria and harvesting a broader number of organs, would allow to investigate the kinetic of the expression of the identified genes throughout the infectious process more precisely.

Furthermore, each of the protein identified in this study is immunogenic and is likely to be involved in the infection process, either by contributing to the bacterium’s virulence or its survival in the host. Therefore, these constitute new possible targets for the development of vaccines and therapeutants against *A. salmonicida*.

## Materials and methods

### Ethics statement

This study was approved by the institutional ethics committee of the University of Veterinary Medicine Vienna and the national authority according to §26 of the Austrian Law for Animal Experiments, Tierversuchsgesetz 2012 – TVG 2012 91 under the No. GZ 68.205/140-II/3b/2012.

### Fish challenges and sampling

Prior to the start of the experiment, specific-pathogen-free rainbow trout (*Oncorhynchus mykiss*) of approximately one year of age, weighting an average of 92 grams, were distributed between 12 aquaria, 15 fish per aquarium. Each of the aquaria was 100 litres in volume and alimented by running water and kept at a temperature of 18°C ±2.

*A. salmonicida* subsp. *salmonicida*, belonging to the isolates A14390 was isolated from a clinical case in rainbow trout in Austria. The bacterium was cultivated on Blood-Agar plate, where it produced the characteristic brown diffusible pigment, as well as Coomassie Brilliant Blue Agar, where it produced characteristic blue colonies [46]. The identification of the bacterium was then confirmed by biochemical profiling followed by Gram strain.

*A. salmonicida* subsp. *salmonidica* was grown overnight in 15 ml of Brain Heart Infusion (BHI) at 15°C. The bacterial concentration within the broth was measured by spectrophotometry and adjusted to one corresponding to a concentration of 10^8^ CFU per ml, based on correspondence curves previously established in our laboratory. Bacteria were diluted in broth to a concentration of 10^3^ Colony Forming Units (CFU) per ml. Cultures were split in 1 ml aliquots, pelleted at 4000 rpm for 10 minutes and re-suspended in 100 μl of 0.9 M sterile phosphate buffered saline (PBS) and transferred into 60 individual 1 ml syringes. Thirty of these syringes were used to challenge fish by intra-peritoneal injections and 30 per intra-muscular injections. Furthermore, 30 fish were challenged by two hours immersion in a solution containing 10^4^ CFU per ml. The fish belonging to each of these routes of infection were divided between 2 aquaria, 15 fish in each aquarium. In addition, for each infection route, 30 negative control fish were mock infected by either injection of a solution of sterile 0.9 M PBS or immersion in a solution of un-inoculated BHI broth. Infection doses were adjusted based on our previous experiments using isolate A14390 and aimed at maximizing the quantity of blood collected while reducing the number of fish used. This was achieved by infecting the fish with the highest possible dose without running the risk of accidentally killing. In all cases, fish were lightly sedated before handling by a brief immersion in an aerated solution of tricaine mesylate (MS222; Sigma) at a concentration of 7 × 10^−4^ g/ml. Fish were returned to their tank immediately after being infected and monitored twice per day.

Subsequently, 3 fish from each treatment and control groups were sampled at the following time points: 1 hour, 6 hours, 12 hours, 48 hours, 3 days, 1 week, 2 weeks, 1 month, 2 months and 3 months post infection. These various sampling points allowed to obtain antibodies from the various phases of the immune response as it is known that the antibody profile can change over time.

Fish were euthanized by prolonged immersion in a solution of MS222 at a concentration of 10^−3^ g/ml and blood was harvested from their caudal veins using heparinised syringes. The spleen, liver and anterior kidneys were then aseptically removed and stored in RNAlater (Sigma) at −80°C. Blood was centrifuged at 4000 rpm for 5 minutes and the supernatant was carefully removed and stored at −20°C until adsorption.

### RNA extraction and cDNA synthesis

RNAs were extracted from the organs sampled at various time points (1, 6, 12, 48 hours and 1 week post infection) using a RNeasy kit (Qiagen). In addition, RNAs were also extracted from the organs of uninfected fish (to act as negative control) and in vitro cultures of *A. salmonicida* subsp. *salmonicida* isolate 14390.

One microgram RNA was used to synthesise cDNA using an IScript kit (Bio Rad) according to the manufacturer’s instruction.

### Estimation of the bacterial abundance in the spleen at various time points

In order to optimize the reverse transcriptase quantitative PCR procedure (RT-qPCR), it was elected to focus on the two time points at which bacterial loads were highest. These two points were identified by conducting real-time PCRs (qPCR) using cDNA constructed from the spleens harvested at various time points as well as primers targeting the bacterial 16S rRNA (Table 2). PCR assays were optimized using gradient PCRs to determine the optimal annealing temperature and primer concentration. A CFX96 Touch Real-Time PCR detection system (BIO-RAD) was used to quantify gene expression levels using iQ SYBR Green Supermix (BIO-RAD). A qPCR was performed in a final volume of 20 μl that contained 4 μl of 1:10 fold diluted cDNA, 0.4 μM of each primer, 1X SYBR Green Supermix as well as sterile distilled water. Trout beta-actin [47] was used as the reference gene for normalization.

**Table 2:** **List of the primers used during this study**

**Primer name:**	**Annealing temperature**	**Amplicon size (bp)**	**Sequence:**
Trout Bact F	53°C	260	ATGGAAGGTGAAATCGCC
Trout Bact R			TGCCAGATCTTCTCCATG
16S rRNA- Forward	57°C	148	ATATTGCACAATGGGGGAAA
16S rRNA- Reverse			GTTAGCCGGTGCTTCTTCTG
UDP- Forward	57.5°C	94	CCGATTATCTCCCGGTTTTA
UDP-Reverse			CCGTTGAGGATCAGGGTAAT
RpoD- Forward	50°C	157	ACAAAGCTCCGTTGCAGAGT
RpoD- Reverse			ACCGATATGGGTAGCAGTCG
Hypothetical protein- Forward	50°C	145	AATCTGCTGTTCGTCGATCC
Hypothetical protein- Reverse			AAAACACGCAGAGCCAGACT
TonB- Forward	55°C	139	GTTGGTGGTCTGGGTACCTG
TonB- Forward			TCAATGACTCACCGGCCAAA

### Construction of the expression library

*A. salmonicida* subsp. *salmonicida* isolate A14390 was grown for 36 hours in 7 ml of BHI at 15°C. Afterward, the bacterium was pelleted by centrifugation at 4000 g for 10 minutes and its genomic DNA was isolated using a Qiagen DNeasy kit (Qiagen) according to the manufacturer’s instructions. The DNA was digested overnight with EcoR1 (Fermentas) at 37°C before stopping the reaction by heating at 65°C for 20 minutes. The digest was run on a 2% agarose gel at 65 V for two hours and a section of the gel, harbouring DNA fragments ranging from 4 kb to 6 kb in size, was excised with a sterile scalpel blade and purified using the Qiaquick gel extraction kit (Qiagen).

The resulting DNA was ligated into an EcoRI pre-digested bacteriophage Zap Express vector (Agilent Technology) according to the manufacturer’s instructions using the DNA ligase kit (Agilent technologies). Afterwards, the resulting DNA was packaged into a viral capsid using Gigapack packaging extract (Agilent) and used to infect XL1-Blue MRF’ *E. coli* grown on NZY (NZ-Amine and Yeast Extract) agar, according to the manufacturer’s instructions. To assess the efficiency of the library, random plaque-forming unites were converted into phagemids and sequenced using the T7 primer to determine the presence of an insert.

### Adsorption of the antibodies

Antibody adsorption aims at exposing the antibodies to the antigens expressed by the bacteria under in vitro culture conditions. The antibodies that recognize such antigens will bind them and be removed from the pool, only leaving behind the antibodies that target proteins that are not expressed under such conditions.

In order to perform adsorption of the antibodies, *A. salmonicida* subsp. *salmonicida* cultures in 20 ml BHI were incubated at 15°C for 48 hours. Afterwards, 20 ml cultures of *E. coli* XL1-Blue-MRF’ in Luria-Bertani (LB) broth, supplemented to 10 mM MgSO_4_ and 0.2% w/v maltose, were inoculated and incubated at 37°C overnight. Protease inhibitor cocktail Set II (Merck) at a dilution of 1:10 was then added to each culture before dividing them into five aliquots of four ml each that were processed in different ways.

Unprocessed bacterial lysate was obtained by sonicating the bacteria on ice using a UW1070 Sonopuls (Bamdelin). Four nitrocellulose membranes of 0.45 μm pore size (Sigma Aldrich) were placed into the lysate and incubated at room temperature for 30 minutes on a rocking table in order to be coated with the proteins it contained. The membranes were then air-dried on Whatman chromatography paper (Sigma Aldrich) at room temperature. Afterwards they were washed 5 times for 5 minutes in 50 ml of Tris-Buffered Saline (TBS) composed of 20 mM Tris–HCl (ICN Biochemicals) and 150 mM NaCl (Sigma Aldrich) and blocked by immersion for one hour in 50 ml of blocking solution (Opti-4CN Substrate kit; Biorad) at room temperature on a rocking table.

The heat-denatured lysate was coated onto nitrocellulose membranes in the same fashion, with the exception that the lysates were heat-denaturated at 95°C immediately after sonication and before immersion of the nitrocellulose membranes.

The whole cell bacteria were obtained by centrifuging two aliquots of each bacterium at 4000 g for 10 minutes and removing the supernatant.

Before starting the adsorption process, antibodies belonging to all time-points and infection routes were tested from reactivity by slide agglutination. Then, reactive antibodies were thawed and pooled and sequentially exposed to the in vitro antigens: The pooled antibodies were incubated on top of each of the lysates-coated nitrocellulose membranes, one after the other, for one hour each at room temperature with gentle rocking. Then, the bacterial pellets were resuspended in the antibodies solution. Incubation lasted one hour at room temperature. Afterwards, the bacteria, and the antibodies that bound them, were removed by centrifugation at 4000 g for 10 minutes.

Following adsorption, the antibodies were diluted 1:500 in antibody dilution solution and stored at 4°C.

To test the efficiency of the adsorption step, whole cells and sonicated lysates from both *A. salmonicida* subsp. *salmonicida* isolate A14390 and *E. coli* were loaded in duplicate and separated by sodium dodecyl sulfate -polyacrylamide gel electrophoresis (SDS-PAGE). The gel was ran at 120 V for 90 minutes and transferred onto polyvinylidene fluoride membranes overnight at 4°C. The next morning, the gel was stained with Coomassie Blue to confirm the efficiency of the transfer. The membrane was cut in two in such fashion that both halve displayed one of each sample. In order to compare the antibody pattern before and after adsorption, both halves were treated either with the adsorbed or the non-adsorbed fish antibodies (1:500). This was followed by a secondary mouse anti-rainbow trout monoclonal antibody (1:200; Aquatic Diagnostic, Stirling). A Horseradish-peroxidase (HRP) conjugated anti-mouse antibody (1:4000; Bio Rad) was then applied followed by colorimetric detection using an Opti-4CN Goat Anti-Mouse Detection Kit (Bio Rad).

### Screening of the genomic library

*E. coli* infected with the viral library were grown on NZY agar plates in order to express the library, as described in Construction of the expression library section. The plates were overlaid with nitrocellulose membranes (pre-soaked in isopropyl β-D-1-thiogalactopyranoside at a concentration of 10 mM) which were left overnight at 37°C to transfer phage particles to the membrane. Afterwards, the plates were stored at 4°C while the membranes were rinsed with TBST (20 mM Tris–HCl, 150 mM NaCl and 0.05% v/v Tween 20) for 5 times 5 minutes. Subsequently, the membranes were covered with blocking solution for one hour at room temperature with gentle rocking before being rinsed two more times for 5 minutes in TBST.

Afterwards, membranes were immersed into 50 ml of the adsorbed fish antibodies (1:500) and incubated overnight at 4°C with gentle rocking before being rinsed for 5 times 5 minutes in 50 ml of TBST. Following this step, the membranes were rinsed before being exposed for 4 hours first to the secondary mouse anti-rainbow trout monoclonal antibody (1:200) then to the HRP conjugated anti-mouse antibody (1:4000) at room temperature with gentle rocking.

The membranes were examined for the positive signals which indicated that one of the remaining adsorbed fish antibodies had reacted with an *A. salmonicida* subsp. *salmonicida* antigen. Because of the initial adsorption process, the corresponding antigens were specifically expressed during the infectious process and not in the in vitro cultures. The signals were marked on the membrane and the portion of the NZY agar from where they originated was cored and sampled.

In addition, viral particles that had generated the positive signals were added on plates inoculated with *E. coli*. The zones of lysis generated by these viral particles were scrapped and deposited in duplicates onto membranes. The membranes were processed following the protocol previously described. Repeated detection of positive signals on these membranes confirmed the accuracy of the initial screening.

### Gene identification

The plaques that corresponded to the positive signals were excised from the agar plate using a sterile scalpel blade. The phage DNA was converted into a phagemid according to the instructions from Agilent Technology.

The bacteria carrying the phagemids were isolated on selective LB agar supplemented with 50 μg/ml of kanamycin and single colonies were inoculated into LB broth similarly supplemented with kanamycin. The broths were incubated overnight at 37°C with rocking at 250 rpm. The next day, the phagemids were isolated using the Qiagen mini-prep kit (Qiagen) according to the manufacturer’s instructions. Inserts were subsequently sequenced by LGC genomics (LGC Genomics, Berlin, Germany) using the T7 and T3 sequencing primers. Identification of the genes was performed by comparison of the sequences against the database from the National Center for Biotechnology Information (NCBI) using Basic Local Alignment Search Tool (BLAST).

### Primer design and confirmatory qPCR

After identification of the genes of interest based on the library screening, a series of RT-qPCR was performed that aimed at investigating the transcription levels of the genes both in vitro cultures and in the bacteria in the organs. It was expected that the specific environment in the organs lead to the upregulation of these genes during the infection process.

Primers were designed using the primer designing tool Primer-BLAST from NCBI (Table 2). Gradient end-point PCRs were used to optimize the annealing temperatures and RT-qPCR was performed on serial dilutions of the template to optimise the reactions for each primer pair.

Afterwards, RT-qPCR was performed to confirm that the genes corresponding to the proteins identified during the membrane screening were indeed upregulated during the infectious process. For technical reasons, we decided to focus on the organs where the bacteria were the most prevalent. These were the organs from the fish infected by intra-peritoneal injection and, based on the results from Estimation of the bacterial abundance in the spleen at various time points section, collected at 6 and 48 HPI.

q-PCRs were performed using the primers specific for our gene of interest as well as the bacterial 16S mRNA. The bacterial 16S mRNA was elected for use as a reference gene based on a previous study conducted in our laboratory where it had successfully served as such [25]. The templates were cDNA from the spleen, liver and anterior kidney samples from infected fish at 6 and 48 as well as cDNA originating from bacteria cultivated in vitro. Alongside these reactions, a series of serial-diluted templates as well as two no-template controls were also included to confirm the efficiency and specificity of the reactions.

C_T_ values were recorded for each reaction and the results were normalized using the bacterial 16S rRNA as the reference gene, as previously described by Menanteau-Ledouble et al. [25]. The mean fold change in gene expression was calculated between the cDNA from the organ samples and that from the *in-vitro* cultures following the 2^-ΔΔ*C*^_T_ method [48]. The 95% confidence intervals were calculated for these mean fold changes. A confidence interval that did not include 1 indicated a significant difference in the gene’s transcription between the organ and in vitro samples.

## Abbreviations

BHI: Brain Heart Infusion
BLAST: Basic Local Alignment Search Tool
cDNA: Complementary DNA
CFU: Colony Forming Unit
GCAT: Glycerophospholipid-cholesterol acyltransferase
HCl: Hydrogen Chloride
HPI: Hours post-infection
HRP: Horseradish-peroxidase
IVIAT: In vivo induced antigen technology
LB: Luria-Bertani
LPS: Lipopolysaccharide
mRNA: Messenger Ribonucleic acid
PBS: Phosphate Buffered Saline
NCBI: National Center for Biotechnology Information
NTC: No template controls
qPCR: Quantitative real-time PCR
RNA: Ribonucleic acid
RpoD: RNA polymerase, sigma D
RT-qPCR: Reverse transcription quantitative real-time PCR
SDS-PAGE: Sodium Dodecyl Sulfate -Polyacrylamide gel electrophoresis
Tricaine mesylate: MS222
UDP-3-O-acyl-N-acetylglucosamine deacetylase: Uridine diphosphate-3-O-acyl-N-acetylglucosamine deacetylase
